# Pediatric sepsis cases diagnosed with group B streptococcal meningitis using next-generation sequencing: a report of two cases

**DOI:** 10.1186/s12879-021-06231-3

**Published:** 2021-06-05

**Authors:** Kazuhiro Horiba, Michio Suzuki, Nobuyuki Tetsuka, Yoshihiko Kawano, Makoto Yamaguchi, Toshihiko Okumura, Takako Suzuki, Yuka Torii, Jun-ichi Kawada, Makoto Morita, Shinya Hara, Tomoo Ogi, Yoshinori Ito

**Affiliations:** 1 Department of Genetics, Research Institute of Environmental Medicine, Nagoya University, Nagoya, Japan; 2 Department of Human Genetics and Molecular Biology, Nagoya University Graduate School of Medicine, Nagoya, Japan; 3 Department of Pediatrics, Nagoya University Graduate School of Medicine, Nagoya, Japan; 4 Department of Pediatrics, Nagoya Memorial Hospital, Nagoya, Japan; 5 Department of Infectious Disease, Nagoya University Hospital, Nagoya, Japan; 6 Department of Pediatrics, TOYOTA Memorial Hospital, Toyota, Japan

## Abstract

**Background:**

Group B Streptococcus (GBS) is an important cause of invasive infection in neonates and infants. Cerebrospinal fluid (CSF) findings and culture may not show evidence of infection early in GBS meningitis. Next-generation sequencing (NGS) has the potential to detect microbial genetic material in patients with infectious diseases. We report two cases of infantile sepsis of GBS meningitis with negative results for CSF culture tests, but positive results for NGS analysis.

**Case presentation:**

Patient 1 was a 22-day-old male infant diagnosed with sepsis and meningitis. His CSF findings showed pleocytosis, decreased glucose, and increased protein levels. However, CSF and blood culture results at admission were negative. He received a total of 3 weeks of treatment with ampicillin and cefotaxime, and showed clinical improvement. GBS was detected through NGS analysis of CSF collected at admission. Patient 2 was a 51-day-old male infant with sepsis. CSF findings on admission were normal, and blood and CSF cultures were also negative. Intravenous ampicillin and cefotaxime treatment were initiated. Treatment was de-escalated to ampicillin alone because *Enterococcus faecalis* was cultured from urine. He was discharged after a total of 1 week of antibiotic treatment. Six days after discharge, he was re-hospitalized for sepsis. Blood and CSF cultures were negative, and *E. faecalis* was again cultured from urine. He received a total of 3 weeks of ampicillin treatment for enterococcal-induced nephritis and did not relapse thereafter. NGS pathogen searches were retrospectively performed on both blood and CSF collected at the first and second admission. GBS was detected in the CSF collected at the first admission, but no significant pathogen was detected in the other samples. Inadequate treatment for GBS meningitis at the first admission may have caused the recurrence of the disease.

**Conclusion:**

Infantile sepsis may present bacterial meningitis that is not diagnosed by either culture testing or CSF findings. NGS analysis for CSF may be useful for confirming the diagnosis of bacterial meningitis.

## Background

Sepsis is one of the leading causes of death among children, and the percentage of all global deaths related to sepsis is highest in children under 1 year of age [1]. Despite its severity, pathogens are not identified in one-third of children with sepsis [2]. Group B streptococcus (*Streptococcus agalactiae*; GBS) is a leading cause of sepsis and bacterial meningitis in infants under 3 months of age. The estimated incidence of GBS infection in infants is approximately 0.5 per 1000 live births [3, 4]. GBS infection that occurs 7–89 days after birth is defined as a late-onset GBS infection. Sixty-five percent of late-onset GBS infection presents as unfocused bacteremia and 20–43% presents as meningitis [3, 5]. Late-onset GBS meningitis is associated with permanent neurologic sequelae in 50% of survivors [6] thus, accurate diagnoses and treatment are required.

Next-generation sequencing (NGS) technology makes it possible to analyze large amounts of nucleic acid sequence data contained in samples in a single assay. Therefore, untargeted metagenomic NGS of clinical samples has been applied for the comprehensive diagnosis of infections, including viruses, bacteria, fungi, and parasites [7]. We previously detected pathogens from blood samples and cerebrospinal fluid (CSF) from patients with sepsis and encephalitis [8, 9]. Here, we report two infants with sepsis that had GBS meningitis revealed through NGS.

## Case presentation

We describe two pediatric cases of late-onset GBS meningitis with sepsis. They were born by vaginal delivery after uneventful pregnancies. Prenatal GBS screening through a vaginal swab was negative in both cases. Sepsis is defined as a systemic inflammatory response syndrome with an infectious disease. Systemic inflammatory response syndrome refers to four parameters: body temperature, tachycardia, hyperventilation, and white blood cell count. Specifically, it is diagnosed when body temperature is > 38.5 °C or < 36.0 °C, or leukocytosis or leukocytopenia is required, and a total of two or more parameters exist [10]. Tachycardia is defined as a heart rate > 180 beats per minute, and hyperventilation is defined as a respiratory rate > 40 per breathes minute (from 1 week to 1 month old) and > 34 breathes per minute (from 1 month to 1 year old).

### Case 1

**Fig. 1:**
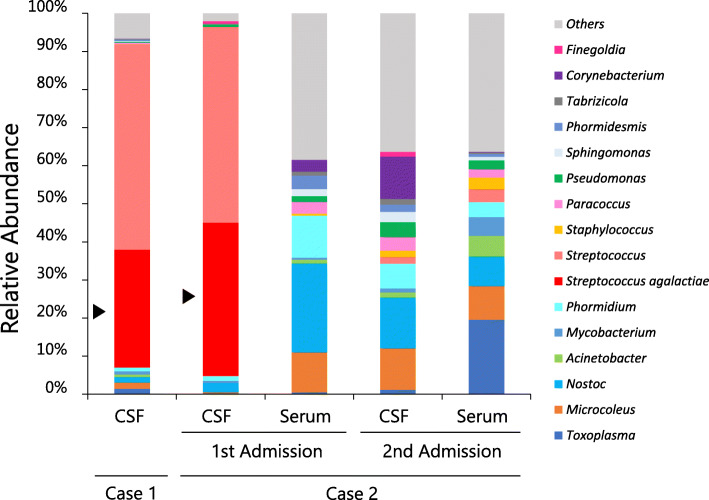
Relative abundance of microorganisms in two infantile sepsis cases. At first admission, *Streptococcus* (the genus level of taxonomic hierarchy) accounted for most of the detected microorganisms in both cases. Furthermore, *Streptococcus agalactiae* (arrowhead) was the most abundant among the *Streptococcus*

A 22-day-old male infant was seen in the emergency room of TOYOTA Memorial Hospital with a 7-h fever history to 38.6 °C and vomiting. He had a bulging anterior fontanelle and peripheral cyanosis. Initial blood examination showed a white blood cell count of 13.1 × 10^3^/μL and a C-reactive protein (CRP) level of 12.0 mg/dL. A lumbar puncture was performed, and examination of the CSF showed pleocytosis (48,896 cells with 91% polymorphonuclear cells), a decreased glucose level of 25 mg/dL, and an increased protein level of 326 mg/dL. He also underwent a sepsis evaluation, including blood and CSF cultures. Leukocytes and bacteria were not seen in Gram staining. He was empirically administered ampicillin (280 mg/kg/day) and cefotaxime (280 mg/kg/day) for bacterial meningitis according to the ESCMID guidelines [11], and intravenous immunoglobulin therapy (1000 mg/kg) was administered on day 2 of admission. The fever broke on day 4 of admission. No conclusive cultures from either blood or CSF were found to guide treatment and de-escalation was not performed. He received a total of 3 weeks of antimicrobial treatment and showed clinical improvement. Magnetic resonance imaging of the brain was performed prior to discharge, and subdural edema and cerebral infarction were noted, but there were no clinical signs. He recovered without any noticeable sequelae after discharge. Later exploratory NGS identified GBS from his CSF collected at admission (Table 1, Fig. 1). The PCR result for GBS was negative using the CSF that showed positive results for NGS (data not shown).

**Table 1:** Next-generation sequencing data of clinical samples

Case No.	Admission	Sample	Total Sequencing Read (reads)	All microorganism Derived Read (reads)	*Streptococcus agalactiae*	
					Number of Reads (reads)	Mapping coverage (%)
1	–	CSF	8,797,854	3754	1078	6.5
2	1st	CSF	181,132	358	135	0.79
2	1st	Serum	155,378	494	0	0
2	2nd	CSF	6,515,464	3348	0	0
2	2nd	Serum	10,209,784	638	0	0

*CSF* cerebrospinal fluid

### Case 2

A 51-day-old male infant presented to the emergency room of Nagoya Memorial Hospital with a 4-h fever and irritability. Upon examination, body temperature was 38.9 °C, pulse rate was 214 beats per minute, respiratory rate was 48 breathes per minute, and SpO2 measured by a pulse oximeter was 99% in room air. Initial blood examination showed leukocytosis (27.4 × 10^3^/μL) and a CRP elevation of 2.13 mg/dL. He also had peripheral cyanosis and was diagnosed with severe sepsis. He underwent a sepsis evaluation, including blood, CSF, and urine cultures. There was no leukocytosis in either the CSF or urine. He was initially administered intravenous ampicillin (300 mg/kg/day) and cefotaxime (300 mg/kg/day). On day 2 of admission, his vital signs normalized. Blood cultures and CSF cultures were negative, while *Enterococcus faecalis* (10^3^ CFU/mL) was detected in urine cultures on day 4 of admission. He received ampicillin (200 mg/kg/day) for 1 week before discharge for enterococcal urinary tract infection. He was discharged after confirmation of a continued normal temperature, no abnormalities on physical examination, and improved blood test results. Six days after discharge, he returned to the hospital with a fever of 39.0 °C, tachycardia, and peripheral circulatory insufficiency. He underwent a sepsis evaluation with lumbar puncture and was empirically administered ampicillin (300 mg/kg/day) and cefotaxime (300 mg/kg/day) for sepsis. The CSF findings were within normal limits. No bacteria were cultured from the blood or CSF. A poorly enhanced area in the kidney was found using contrast-enhanced computed tomography (CT) on the day after admission. In addition, 10^3^ CFU/mL *E. faecalis* was detected in urine culture, and ampicillin (200 mg/kg/day) was administered for enterococcal bacterial nephritis for 3 weeks. Voiding cystourethrogram revealed Grade 4 reflux findings on the right side strongly indicating bacterial nephritis. There was no recurrence of the disease. NGS analysis was performed retrospectively on serum and CSF collected at the first and second admission to detect pathogens. GBS was detected in the CSF collected at the first admission, but no significant pathogen could be detected in the other samples (Table 1, Fig. 1). The PCR result for GBS was negative using the CSF that showed positive results for NGS (data not shown).

### Sample collection and preparation of sequencing

Serum and CSF were collected under aseptic conditions. DNA was extracted from 140 μL serum and CSF, using the QIAamp UCP Pathogen Mini Kit (Qiagen, Hilden, Germany). DNA sequencing libraries were prepared using a Nextera XT library Prep Kit (Illumina, San Diego, CA, USA) according to the manufacturer’s instructions with slight modification [8]. An Agilent 2100 Bioanalyzer (Agilent, Santa Clara, CA, USA) and a QX200™ Droplet Digital PCR System (Bio-Rad, Richmond, CA, USA) were used for the quantification of NGS libraries. Sequencing was performed using the Illumina HiSeqX.

### Data analysis

Sequence data were processed using the metagenomic analysis pipeline PATHDET version 1.0 [12] to detect pathogen-derived sequences. PATHDET reported the sequence-derived pathogen based on previously established threshold criteria [8].

### GBS detection by PCR

PCR using the GBS-specific primers Sag59 (5′-TTTCACCAGCTGTATTAGAAGTA-3′) and Sag190 (5′-GTTCCCTGAACATTATCTTTGAT-3′) was performed for both clinical samples according to methods described previously [13].

## Discussion and conclusions

Infantile sepsis is occasionally accompanied by bacterial meningitis; however, meningitis is not easy to diagnose in some patients. Bacterial meningitis in the early stage of the disease onset of late-onset GBS infection tends to result in normal CSF findings and is negative for CSF culture testing due to the small number of bacteria [14]. Since both patients visited hospitals immediately after fever onset, bacterial pathogens may not have been detectable in culture. Both patients were treated with empiric antimicrobial treatment for GBS infection according to the ESCMID guidelines [11], and the drug susceptibility of GBS in Japan was estimated based on a previous study [15]. The recommended duration of antimicrobial therapy is 14 to 21 days for GBS meningitis [16]. In Case 1, the empiric treatment corresponded to GBS meningitis because the treatment was continued for 21 days. In contrast, ampicillin treatment was administered for only 7 days at the first admission in Case 2. This treatment period may have been inadequate, resulting in the second GBS infection. The urinary tract infection was mainly responsible for the disease condition in Case 2 because the patient had a vesicoureteral reflux complication. However, GBS meningitis may have been at an early stage in its clinical course at the time of the first admission. The number of NGS that detected GBS detected by NGS reached diagnostic levels, but were relatively low [8]. The mother of patient 2 did not carry GBS, but the risk of late-onset GBS disease exists because nosocomial cross-infection is an important source of GBS in late-onset disease [17].

NGS analysis of CSF is advantageous for diagnosing GBS meningitis in pediatric patients with negative CSF cultures. According to a study by Wilson et al. [18], NGS identified pathogens in CSF from 13 (22%) of 58 patients with meningitis/encephalitis that were not identified by conventional clinical testing. PCR is also used as a molecular diagnostic method and has advantages, in terms of sensitivity, for diagnosing meningitis in patients with negative CSF culture [19]. NGS appears to be more suitable as a diagnostic procedure, because it does not rely on the pre-selection of targeted pathogens, but rather is able to detect many potential infectious agents in a single assay. NGS analysis is expected to become a standard diagnostic test for CSF samples, replacing conventional diagnostic procedures. In the pediatric field, NGS analysis that uses only small sample volumes is advantageous because sample volumes are often limited. There was a discrepancy in the results between NGS and PCR in the present cases; the NGS results for GBS were positive and the PCR results were negative. A previous report described the diagnosis of neurological infections using NGS with CSF samples; however, this report did not compare with the results using PCR methods [20]. The size of the amplified DNA was similar in this study; however, the efficacy of amplification may be influenced by unknown factors. Additionally, the region amplified by PCR was not covered by NGS. The difference in sensitivity may be due to differences in the amplification efficiency at different locations of the genome.

In Case 2, the NGS result for GBS was positive in CSF; however, the result was negative in blood. A previous report stated that blood cultures are positive in at least half of patients with bacterial meningitis [21, 22]. Other studies have reported that a substantial proportion (33 to 53%) of neonates with culture-proven meningitis have negative blood cultures [23]. The amounts of bacteria may be skewed in different types of clinical samples. Another possibility is that the efficiency for growth is different between CSF and blood samples. With regard to NGS, the huge amount of host-derived nucleic acids prevents detection of microorganism-derived sequences. In most cases using plasma samples, less than 1% of extracted DNA originates from microorganisms during blood-stream infection [8]. Moreover, NGS analysis using the blood of septic patients requires further efforts to remove human cell-free DNA because this may increase when systemic inflammation is progressing due to sepsis [24]. This study had a few limitations. First, we only experienced two cases. The number of cases assessed using NGS should be increased. Second, the diagnostic tests could not be repeated to confirm negative results because of the small number of CSF samples.

In conclusion, we report two cases of GBS meningitis that were revealed retrospectively by NGS analysis. We also showed that GBS meningitis was lurking in pediatric sepsis negative for CSF culture testing with normal CSF findings. NGS may be useful as a diagnostic tool in the specific clinical settings presented in the present report.

## Data Availability

The data that support the findings of this study are available from the corresponding author upon reasonable request. Sequencing reads generated in this study were deposited at the DDBJ Sequence Read Archive under the following accession number: DRX 241918–241922.

## Abbreviations

GBS: Group B streptococcus
NGS: Next-generation sequencing
CSF: Cerebrospinal fluid
CT: Computed tomography
